# Reticulated Platelets—Which Functions Have Been Established by In Vivo and In Vitro Data?

**DOI:** 10.3390/cells10051172

**Published:** 2021-05-12

**Authors:** Muataz Ali Hamad, Nancy Schanze, Nicolas Schommer, Thomas Nührenberg, Daniel Duerschmied

**Affiliations:** 1 Department of Cardiology and Angiology I, Heart Center, Faculty of Medicine, University of Freiburg, 79106 Freiburg im Breisgau, Germany; nancy.schanze@universitaets-herzzentrum.de (N.S.); nicolas.schommer@universitaets-herzzentrum.de (N.S.); daniel.duerschmied@universitaets-herzzentrum.de (D.D.); 2 Spemann Graduate School of Biology and Medicine (SGBM), University of Freiburg, 79104 Freiburg im Breisgau, Germany; 3 Faculty of Biology, University of Freiburg, 79104 Freiburg im Breisgau, Germany; 4 Department of Cardiology and Angiology II, Heart Center, Faculty of Medicine, University of Freiburg, 79189 Bad Krozingen, Germany; thomas.nuehrenberg@uniklinik-freiburg.de

**Keywords:** reticulated platelets, immature platelets, immature platelet fraction

## Abstract

Reticulated platelets (RP) are the youngest platelet fraction released into the circulation. These immature platelets have increased RNA content, a larger cell volume, more dense granules, higher levels of surface activation markers and are thought to be more reactive compared to their mature counterparts. RP have been associated with cardiovascular disease, diabetes and increased mortality. Yet only a few animal studies investigating RP have been conducted so far and further investigations are warranted. Established methods to count RP are flow cytometry (staining with thiazole orange or SYTO13) or fully automated hematology analyzers (immature platelet fraction, IPF). IPF has been established as a diagnostic parameter in thrombocytopenia, cardiovascular disease and, in particular, the response to antiplatelet therapy. This review seeks to provide an overview of the key features of RP as well as preanalytical and analytical aspects that need to be considered when working with this platelet population.

## 1. Introduction

Platelets are anucleate cell fragments derived from megakaryocytes (MK) in the bone marrow (BM) at a range of 150,000 to 400,000 cells/μL and play significant roles in hemostasis, thrombosis, and inflammation [1]. Reticulated platelets (RP) are the youngest platelets released into the circulation and contain a residual amount of megakaryocyte-derived RNA [2]. RP thus have increased RNA content compared to mature platelets and are characterized by a larger cell volume, more dense granules and higher levels of surface activation markers. They are considered to show increased reactivity and are associated with impaired response to antiplatelet therapy [2,3,4]. Higher levels of RP have been linked to a higher risk of major adverse cardiovascular events [5,6] and are associated with a higher risk of death in patients with acute coronary syndrome [7]. Platelets are refractory to many techniques that are commonly used in cell culture, which increases the need for animal models to manipulate and study platelets in different disease settings. The mouse model has been one of the most commonly used animal models to investigate platelets and serves to imitate a variety of pathological conditions. Murine and human platelets are functionally very similar with some differences in structure. While the usefulness of mouse models is clear, some inherent differences must be acknowledged and appreciated in the interpretation of the data. Here we will focus on the main considerations when studying murine platelets with special regard to the translational aspects of RP research.

## 2. Physiology of Reticulated Platelets

Blood platelets are derived from bone marrow megakaryocytes via a process of MK cytoplasm remodeling which works in a largely similar manner in humans and mice. However, mouse MK contain more prominent demarcation membranes, consisting of supposedly preformed platelet territories [8]. The compartmentalization within the α-granules of both human and murine platelets seems to be very similar which should be seen as further evidence that mouse platelets can mimic processes also present in human platelets [8]. Similarities between mouse and human platelets prevail, but a few distinctions should be acknowledged. In terms of hemostasis, one of the major differences is the platelet count [9,10]. On average murine platelet counts range three times higher than human platelet counts with variations across different mouse strains (average platelet count in mice is 1000–1500 × 10^3^ cells/µL) [11]. Also, mouse platelets are smaller compared to human platelets (mean platelet volumes 4.7 ± 0.3 fl in mice vs. 7.5–10 fl in humans) [12]. Besides these structural differences, the circulating life-span of mouse platelets is markedly shorter than that of human platelets (3–4 days in mice vs. 8–12 days in humans) [13]. Since part of the murine thrombopoiesis takes part in megakaryocytes in the spleen, this could further add to the higher platelet turnover observed in mice [14]. As RP comprise the youngest platelets in the circulation, they are referred to as ‘reticulated’, analogous to reticulocytes in erythropoiesis [15]. In the bone marrow, RP are about 2–3 times higher than in peripheral blood [16]. Through the higher amount of RNA in RP one can distinguish them from mature platelets using adequate staining and analyze them in a quantitative manner [14]. RP are present for ≤ 24 h in humans, whereas in mice they were identifiable for 1.5 days [17]. While in humans, RP account for about 5% of the total platelet population in a steady-state [18], in mice they make up around 7% of the total platelet count [19]. In rats, one study even showed a mean RP percentage of 10% [17]. Mean platelet volume (MPV) is a measure of platelet size. RP are larger than mature platelets, as shown by animal studies as well as in humans after chemotherapy [20,21,22]. It is important to notice that these measurements were done under conditions with increased platelet turnover, during which the ploidy of megakaryocytes increases [23], leading to the observation of increased platelet size together with the ploidy of megakaryocytes [24] and supporting the concept of larger young platelets when turnover increases.

**Figure 1:**
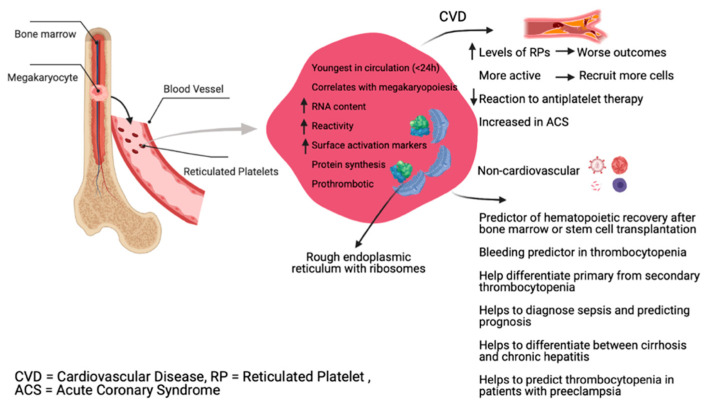
RP properties and roles in different diseases.

On the other hand, platelet size may not correlate with platelet age under steady state platelet production and clearance. A study of healthy adults using the Abbott Sapphire analyzer highlighted a negative association between RP and MPV [25], suggesting that an increased platelet size does not correlate well with platelet age under steady state platelet generation in humans. When platelet turnover increases, larger young platelets are produced, likely because alternative pathways of platelet production are triggered [26]. Although RP might be larger in size than mature platelets, the morphological properties of RP are difficult to observe mainly due to the lack of suitable markers and their relatively low presence in total platelets [18]. Figure 1 summarizes different RP properties and roles.

## 3. Pre-Analytical Aspects

Platelets can be easily activated; hence to perform accurate in vitro platelet assays it is important to ensure that sample collection does not lead to platelet activation. The small size of mice and their limited blood volume highly increase the chance of platelet activation during blood withdrawal. Blood can be collected using different methods in mice and choosing the right or suitable route depends on different factors such as the amount of blood to be collected, the frequency of blood collection and the size or nature of the collection tool. Blood can be collected from the retrobulbar venous plexus, jugular vein, carotid artery, inferior vena cava, heart, tail, or lateral saphenous veins (Table 1).

The blood volume of an average mouse (~25 g) is ~1.8 mL, and adult mice tolerate the withdrawal of up to 0.7 mL and recover their normal blood volume within 24 h [27]. Blood is collected into a tube containing an anticoagulant and all the anticoagulants used in human platelet studies have been successfully used for mice platelets. Choosing the appropriate agent depends on the ultimate goal of the platelet study involved. Blood collection in mice is usually done under general anesthesia, which can be achieved using different agents and routes [28]. The commonly used routes are intraperitoneally (sodium pentobarbital, ketamine, avertin) or inhalation (isoflurane) [28]. Even though no reports show any effect of anesthesia on platelet function in mice, parenteral anesthesia has been shown to decrease platelet aggregation and inhibit the release reaction in both humans and animals [28]. Thus, anesthesia with isoflurane should be the preferred mode for the collection of platelets in mice. As for other hematological parameters, RP/IPF are normally measured in blood samples collected into K2-EDTA. Citrate-theophylline-adenosine-dipyridamole solution (CTAD) might be preferred over EDTA in some cases, such as when using a Sysmex XE-5000, as a study reported more stable IPF in blood from patients with chronic ITP [29]. Stability and storage temperature before analysis is method dependent but in general, a slight increase occurs during 24 h in EDTA-anticoagulated blood samples at room temperature. Using different automated analyzers, the values in human blood were stable within 6 h after blood collection at room temperature [30], while for Mindray BC-6800 stability was shown within 8 h [31]. When platelets collected from one animal are not sufficient in number, it may be necessary to collect blood from multiple animals [5,6,7,8,9,10,11,12,13,14,15] for one experiment. Platelets are isolated from whole blood for most of the assays in the form of platelet-rich plasma (PRP). The preparation of PRP can be done in different ways; either using one-step centrifugation, or sequential centrifugation of plasma and buffy coat [32]. PRP usually still contains a few leukocytes which contaminate some assays, such as RNA-Seq of platelets for which leukocyte depletion should be performed. This can either be achieved by magnetic beads covalently coupled to anti-CD45 antibodies or by filtration through specific membranes. For platelet research, certainly the magnetic ‘no-touch’ techniques are preferable to avoid undesired platelet activation.

### Pathogens

To ensure reproducibility of research it is required to have laboratory animals free of disease and other conditions that could interfere with the observed outcomes. Mice are prone to infections and these infections even if they are subclinical may influence animals’ physiology and immunity which in turn might have effects on platelets. A good example of these pathogens is *Helicobacter hepaticus* which is a known ‘unwanted’ pathogen present in rodent colonies [33]. A recent study from the University of Rio de Janeiro for instance examined mice from their animal facilities for *Helicobacter* spp. showing that ~59% of fecal and ~70% of intestinal samples were positive for this pathogen [33].

## 4. Methods for Reticulated Platelets Determination

### 4.1. Flow Cytometry

The most commonly used method to evaluate RP is flow cytometry (FCM) with nucleic acid binding fluorochromes such as thiazole orange (TO) or SYTO13. The higher RNA content in RP makes it possible to discriminate these from mature platelets when staining against RNA with a nucleic acid-specific dye. TO is such a nucleic acid-specific dye that exhibits several thousand-fold increases in fluorescence emission upon binding to RNA or DNA [34]. TO is excitable at 488 nm which makes it suitable for most flow cytometers, easily permeates the cell membrane, and has a high quantum yield when bound to nucleic acids. When used in RP studies, there is a direct dose–response relationship between the amount of TO added and the number of RP observed, reaching a plateau at 5 μg TO per 5 μL of whole blood, while 12 μg was found to be the optimum [19]. Increasing the amount of TO would produce a population shift or mean channel shift that needs to be accounted for when setting gates for TO positive events [19]. Using a platelet-specific antibody (such as CD41, CD61) combined with TO is therefore an accurate method of counting RP.

The nucleic acid dye SYTO 13 is an alternative for staining RP which proves to have several advantages over TO [35]. SYTO13 shows high stability over time, facilitating extended experimental analysis (for instance making it more suitable for sorting) and higher quantum yield. For both dyes, it is important to note that nonspecific labeling may occur by staining of mitochondria or dense granules with both dyes.

### 4.2. Fully Automated Analyzers 

Besides FCM, automated cell counters have been developed to measure human RP as a fraction of the total platelet count such as the Sysmex hematology analyzer, Abbott CELL-DYN Sapphire, and Mindray analyzer.

#### 4.2.1. Sysmex Analyzers

The Sysmex XE-2100 and 5000 hematology analyzers allow the counting of RP together with reticulocytes using dedicated software and fluorescent dye (polymethine). The immature platelet fraction (IPF) is expressed as both a percentage of total platelets (%IPF) and in the absolute count (#IPF). The new generation of the Sysmex device (XN) uses a specific platelet channel (PLT-F), and a different fluorescent dye (phenoxazine) [36]. Some studies have compared the two generations XE and XN and concluded that the XN generation has higher and broader reference intervals of %IPF [37,38,39]. The %IPF in adult, healthy humans range from 0.7–10.1 with some sex-specific reference intervals [22,23,24]. The concept that both generations of analyzers use is the measuring of forward scatter (cell volume) and fluorescence intensity (RNA content), and a computer algorithm discriminates between the mature and immature platelets on these bases.

#### 4.2.2. Abbott Analyzer

The Abbott CELL-DYN Sapphire is a hematology analyzer capable of measuring RPs. The measurement of RP is based on the fluorescent dye CD4K530 as part of the reticulocyte assay. The platelets are separated from the red blood cells by recording three angles of scattered light plus fluorescence. The algorithm used defines RPs in a scatterplot of FL1 versus light at a 7° angle [25].

#### 4.2.3. Mindray Analyzer

The Mindray BC-6800 measures IPF by asymmetric cyanine-based dye for staining RNA. IPF is expressed as a percentage and is derived from forward scatter vs. sideward fluorescence scatterplot. The IP/RP can be reported as an absolute number (×10^9^/L) calculated by multiplying the platelet count by the value percentage [31].

RP are often investigated in more detail using flow cytometry. IPF is obtained with automated hematology analyzers. The term “young platelets” is more descriptive. These values when compared show a modest correlation with a similar but numerically different trend. The different results obtained with different methods may be attributed to several analytical and pre-analytical reasons. The used stains (solution ready to use or home-preparation), contamination from other blood cells such as leukocyte or the unspecific binding of the dyes and, the different gating strategies are all possible explanations for the differences between the results obtained with different methods. For instance, the flow cytometer gate is usually set at 1%, while for Sysmex it is proprietary and not modifiable [31]. Therefore, although the terms RP and IPF are frequently considered synonymous, in practice the two parameters cannot be used interchangeably and it is important to distinguish between them as they only partially overlap [30].

In basic research, if mouse platelets are analyzed using automated cell counters that are designed for human platelets, it is necessary to adjust the discriminators due to the smaller size. Very low platelet counts might be attributed to improper blood collection or improper adjustments on the automated cell counters. Another variable that might affect platelet counting is the presence of cellular fragments with a size similar to platelets.

## 5. In Vivo Data

### 5.1. Animal Studies

#### RP in Diabetes and Increased CVD Risk

Diabetes is one of the major risk factors for cardiovascular disease (CVD) [40], with atherosclerosis as a major driver [41,42]. Yet the underlying mechanisms of how hyperglycemia accelerates atherogenesis still remain widely unresolved. Given that reticulated platelets act in a hyperreactive manner and have been associated with increased CVR, it is of great importance to investigate the role of these immature platelets in diabetes. In one study, diabetes was induced in C57BL6 mice using streptozotocin, a toxin which destroys the beta cells in the pancreas [43]. Interestingly these diabetic mice had increased RP, platelet-leukocyte aggregates, and elevated leukocyte activation and proliferation. Increased production/proliferation of platelet progenitors is associated with diabetes resulting in elevated circulating levels of highly activated RP. To take a closer look at the reason behind such findings, the livers of diabetic mice were examined. The diabetic mice were found to have increased Kupffer cell population in the liver and a high proportion of these cells produce interleukin 6 (IL-6), a proinflammatory cytokine. Increased IL-6 production was a response to hyperglycemia as the neutrophil-derived S100 calcium-binding proteins A8/A9 (S100A8/A9) interact with the receptor of advanced glycosylation end products (RAGE) on hepatic Kupffer cells. IL-6 increases thrombopoietin which in turn interacts with platelet progenitor cells leading to increased RP production. This hypothesis was tested by either chemical depletion of Kupffer cells or genetic knockout of IL-6 resulting in reduced RP in the diabetic mice. Obese mice (diet-induced or due to genetic background (*ob/ob*) had more IL-6 production by Kupffer cells and more RP than lean mice. To test whether hyperglycemia is responsible for these findings, diabetic mice’s blood glucose levels were reduced. This resulted in normalization of RP levels and Kupffer cells produced lower amounts of IL-6. Depleting neutrophils or Kupffer cells, or inhibiting S100A8/A9 binding to RAGE, reduced diabetes-induced thrombocytosis. The authors then examined some of these parameters in a cohort of people with type 2 diabetes. Patients with diabetes and peripheral vascular disease taking aspirin had increased levels of RP and platelet-monocyte-aggregates compared to healthy controls taking aspirin. The authors concluded that glycosylated hemoglobin, as well as increased plasma levels of S100A8/A9 correlate with reticulated thrombocytosis in DM-2 patients. These findings provide insights into the mechanisms that regulate platelet production and may help to improve current antiplatelet therapies.

### 5.2. Clinical Utility of RP

The immature platelet fraction (IPF) or RP has numerous clinical applications in diagnosing and monitoring different diseases. The clinical applications of IPF and RP are summarized in (Table 2).

#### 5.2.1. Thrombocytopenia

The causes of thrombocytopenia are diverse and sometimes difficult to confirm. The main question in any thrombocytopenia case is the determination of the underlying cause: is it related to bone marrow failure or peripheral causes? RP/IPF is a useful parameter in differentiating between thrombocytopenia due to bone marrow failure in which the proportion of RP is within the reference range, or thrombocytopenia due to peripheral destruction or acute blood loss in which the proportion is increased [30,44,45,46,47,48]. A strong correlation was detected between platelet count and IPF in primary immune thrombocytopenia (ITP). In ITP patients, the median IPF value ranges from 5.7–22.3%. In aplastic anemia (AA) with an isolated thrombocytopenia presentation, IPF has been shown to increase [37]. The sensitivity and specificity of %IPF in differentiating between ITP and AA are 54% and 92%, respectively, with %IPF of 7.3% as the best cut-off [37]. In chemotherapy patients who had fewer platelet transfusions, an increase in IPF occurred after 2–3 days indicating platelet recovery [62].

The increase in IPF is a crucial observation that can prevent unnecessary platelet transfusion and may serve as an indicator for successful engraftment after stem cell transplantation [63,64,65,66].

#### 5.2.2. Myelodysplastic Syndromes

IPF is also a useful marker in some myelodysplastic syndromes, as a high IPF is associated with poorer prognosis due to megakaryocytic dysplasia or karyotypic abnormalities [49,50].

#### 5.2.3. Cardiovascular Disease and Antiplatelet Therapy

High IPF in patients with coronary artery disease has been reported in several studies, especially with acute coronary syndrome (ACS) [51,67,68,69,70]. More specifically, higher levels of RP have been linked to a higher risk of major adverse cardiovascular events [51,68,69,70,71] and are associated with a higher risk of death in patients with ACS [72]. In patients with ST segment elevation myocardial infraction (STEMI), RP were increased four-fold compared to control patients [73]. Not only in relation to healthy controls, another study showed that RPs were particularly elevated in STEMI patients compared to other types of acute coronary syndromes [70]. An interesting aspect in which RP might have a significant role is myocardial ischemia/reperfusion (I/R) injury, which is the tissue damage caused when blood supply returns to tissue after a period of ischemia. I/R injury is caused by the interventional reopening of an occluded coronary vessel in the context of MI. The platelet population as a whole during I/R has been investigated in the past, but the role of platelet subpopulations still remains unclear. The response of RP to antiplatelet therapy is of great interest, because RP seem to exhibit resistance to common antiplatelet therapies at least to some extent [74]. In ACS, patients received antiplatelet therapy with aspirin and P2Y_12_ antagonists (clopidogrel/prasugrel or ticagrelor) as a mono- or dual-therapy for the prevention of thromboembolic complications. It is uncertain whether the reduced drug effect is due to a stronger RP reactivity or due to an increase in newly formed platelets.

#### 5.2.4. Infection Diagnosis and Control

A relationship between IPF and infection has been reported. IPF was found to be a useful tool in detecting an infectious state, and it was sensitive enough to differentiate a serious from a minor infection [55]. Depending on the patients’ clinical condition, IPF varied, and values below 5.5% showed normalization of body temperature by 2–7 days [55]. In sepsis, a significant correlation between higher IPF and the diagnosis of sepsis was established [57]. A decrease in IPF# was an independent predictor of severe thrombocytopenia and mortality [57]. Other researchers have concluded that IPF could predict sepsis development before symptoms appear [56]. Another interesting finding was that the HIV viral load correlated with IPF, which might be attributed to a secondary HIV-driven platelet activation [75]. IPF can also predict platelet recovery 24–48 h earlier in dengue infection [76].

#### 5.2.5. Pregnancy Complications

Pregnancy is associated with an increase in IPF, usually between 20 and 40 weeks of gestation. An increase in IPF of more than 7.5% suggests increased thrombopoiesis in response to increased platelet consumption [77]. IPF values are significantly higher in patients with gestational hypertension when compared to normotensive pregnancy [58,78]. Changes in IPF may be predictive of the development of thrombocytopenia in patients with preeclampsia, as there is a correlation between higher IPF and lower platelet count [58].

#### 5.2.6. Liver Diseases

Hepatitis B or C patients who are thrombocytopenic have elevated IPF when compared to nonthrombocytopenic infected patients [79,80]. On the other hand, patients with liver cirrhosis have higher IPF values than patients with chronic hepatitis [59,60,61], thus allowing for differential diagnosis between cirrhosis and chronic hepatitis.

## 6. In Vitro Data

High on-treatment platelet reactivity and reoccurrence of thrombotic events represent a major therapeutic hurdle when attempting to prevent thrombotic complications. Besides several pathological states linked to high on-treatment platelet reactivity, an increased rate of platelet turnover is a major contributing factor. A study in which the interactions of populations of platelets from healthy volunteers and patients with stable cardiovascular disease were followed in vitro showed an overproportion of RP in the core of the aggregates being formed [54]. These findings indicate that RP are more reactive, have a greater propensity for recruitment to thrombi, and “act as seeds” for platelet aggregate formation [60]. More importantly, this phenomenon was particularly observed in samples from patients treated with aspirin plus a thienopyridine, but was absent in samples taken from patients treated with aspirin plus ticagrelor. Another study to investigate the influence of RP on cangrelor and transitioning strategies to oral P2Y12-receptor inhibitors found a detectable correlation between RP and platelet reactivity in patients receiving thienopyridines, but not ticagrelor [52]. During treatment with aspirin plus thienopyridine, the short pharmacokinetic half-lives of these drugs may explain this phenomenon, while during treatment with ticagrelor, which has a longer half-life and ability to act as a circulating inhibitor, this phenomenon is absent. This suggests that newly formed and somewhat drug-resistant RP play a key role in limiting the effectiveness of antiplatelet therapies.

## 7. Conclusions

In conclusion, the youngest RPs in the circulation are more reactive and show greater tendency to recruit other platelets and immune cells to the site of injury. Containing residual RNA makes it possible to stain RPs and differentiate them from reticulocytes and mature platelets. RPs have been associated with higher CVR in ACSs, major adverse outcomes and, most importantly, increased mortality. Not only do they spike in STEMI and all forms of MI, but diabetes also promotes reticulated thrombopoiesis. Additionally, IPF is utilized as a disease and prognosis modifying parameter in a variety of different conditions. Whether RP/IPFs only serve as prognostic markers or whether RPs themselves are drivers of disease and pose a targetable threat still needs to be investigated in depth. Only a few animal studies on RP have been carried out to this day and most of our current knowledge is derived from observational studies in humans. Although mouse platelets are in many ways similar to those of humans, there are still some questions to be raised when using murine platelets, for example, whether platelets isolated from different sites are similar or whether the method of collection can influence platelet activation. Further considerations limiting research reproducibility are potential differences across strains or the presence of undesirable pathogens. Nevertheless, mouse models have successfully mimicked human diseases and have provided insights into their underlying pathology. Interpreting the results as relevant to a specific experimental setting is crucial and there is no ‘best’ model for every study.

## Figures and Tables

**Table 1:** Different blood collection routes from mice.

Route	Blood Volume	Collection Tool	Endpoint	Notes
Retrobulbar venous	~0.5 mL	~1.5 cm long glass capillary.	Sacrifice the animal.	Blood contact with glass can activate platelets.
Cardiac puncture	~1 mL	21 G needle and a syringe.	Sacrifice the animal.	Tearing of the heart muscle can lead to thrombin generation and platelet activation.
Inferior vena cava	~1.2 mL	22–27 G needle and a syringe.	Sacrifice the animal.	This method probably results in the least platelet activation.
Tail veins	Up to ~50 µL	Horizontal incision in the tail vein.	Suggested for multiple blood collection.	‘Milking’ the tail should be avoided to avoid higher erythrocyte and leukocyte count in the sample.

**Table 2:** Clinical applications of IPF and RP.

Proposed Clinical Applications	Intended Goal	Reference
Thrombocytopenia	Differentiating platelets hypoproduction from accelerated destruction	[44,45,46,47,48]
Bone marrow/stem cells transplantation or chemotherapy	Predicting platelet recovery	[49,50,51,52,53]
Myelodysplastic syndromes	Clinical evaluation and assessing prognosis	[49,50]
Cardiovascular diseases	Assessing the role of platelet activation in prognosis	[51,52,53]
Antiplatelet therapy	Predicting treatment response	[52,54]
Infectious diseases	Early diagnosis	[55]
Sepsis	Predicting sepsis in critically ill patients	[56,57]
Pregnancy complications	Monitoring preeclampsia	[58]
Liver diseases	Differential diagnosis between cirrhosis and chronic hepatitis	[59,60,61]
